# 161. Impact of Committee-Based Diagnostic Stewardship on Performance of metagenomic Next-Generation Sequence Testing for Suspected Infections in Children

**DOI:** 10.1093/ofid/ofad500.234

**Published:** 2023-11-27

**Authors:** Brian S Allen, Paul K Sue, Laura Filkins, Mehgan Teherani

**Affiliations:** University of Texas at Southwestern (UTSW), Grapevine, Texas; UT Southwestern Medical Center, Dallas, Texas; University of Texas Southwestern/Children's Health, Dallas, Texas; UTSW Medical Center Dallas, Dallas, Texas

## Abstract

**Background:**

Metagenomic next generation sequencing (mNGS) is a novel, unbiased approach to identify clinically relevant microorganisms. While an increasing body of lliterature suggests the potential positive impact of mNGS testing, best–use cases remain unclear, with rates of clinically significant outcomes as low as 10%. We implemented a restrictive testing model requiring pre-approval for mNGS testing via an ad-hoc committee of infectious disease specialists and pathologists, to optimize utilization of mNGS testing and aid in interpretation of results. We hypothesized that implementation of this diagnostic stewardship model would lead to higher rates of clinically actionable results.

**Figure:**
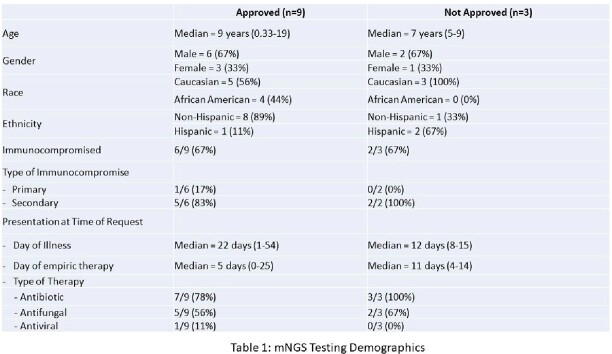

**Methods:**

We performed a retrospective review of mNGS requests at our institution from August 1, 2018 – April 30, 2021. Cases were evaluated by the mNGS committee within 24 hours, and following committee-provider discussion, either approved, denied as inappropriate, or denied in favor of additional conventional evaluation. Charts were subsequently reviewed and mNGS testing adjudicated as having either positive, negative, or no impact .

**Results:**

12 mNGS requests were adjudicated, with 9 subjects approved for testing. Median age of participants was 8 years, with a large proportion (67%) of immunocompromised individuals. The most common indication for mNGS testing was persistent fever (58%). All samples were collected within 24 hours of request, with a median result time of 4 days. Among the 9 approved tests, seven (67%) were deemed to be clinically significant and directly impacted care, while three (33%) resulted in no impact. In total, mNGS testing led to 7 changes in management, 3 clinically relevant organisms identified, and 2 invasive procedures avoided. No adverse outcomes were observed among the denied requests. .

**Conclusion:**

Plasma cell-free DNA mNGS is a promising albeit limited diagnostic tool for clinically relevant infectious diseases, and in the absence of diagnostic stewardship, may lead to unnecessary treatment and excess costs. Implementation of committee-based pre-approval and testing restriction led to high rates of clinically actionable results, and may improve the utility of mNGS testing, while neither delaying care nor negatively impacting outcomes.

**Disclosures:**

**Paul K. Sue, MDCM**, Allovir, Inc: Participant in Industry Sponsored Trial|Gilead Sciences, Inc: Participant in Industry Sponsored Trial|Merck & Co.: Participant in Industry Sponsored Trial **Laura Filkins, PhD**, Avsana Labs: Board Member|Avsana Labs: Stocks/Bonds|Biofire Diagnostics: Grant/Research Support

